# What Do Healthcare Providers Advise Women with Multiple Sclerosis Regarding Pregnancy?

**DOI:** 10.1155/2014/819216

**Published:** 2014-03-05

**Authors:** Annette Wundes, Roxanna N. Pebdani, Dagmar Amtmann

**Affiliations:** ^1^Department of Neurology, Multiple Sclerosis Center, University of Washington, McMurry Building, 1536 N. 115th Street, Seattle, WA 98113, USA; ^2^Department of Rehabilitation Medicine, University of Washington, Box 359612, Seattle, WA 98195, USA

## Abstract

Pregnancy in multiple sclerosis (MS) is considered safe for both the woman and the child. Nevertheless, pregnancy issues in MS are complex both from a patient's and a provider's perspective. In an anonymous survey, 28 healthcare providers in the United States reported on the management of multiple sclerosis (MS) during pregnancy. Participants were asked about their recommendations to patients about the use of disease modifying therapies during pregnancy and breastfeeding and general recommendations about MS and pregnancy. Healthcare providers were also asked about sources from which they receive information about the management of patients with MS. Results suggested that healthcare providers do not discourage pregnancy for women with MS, recommend that women not use disease modifying therapies while pregnant, and have a positive view of breastfeeding for women with MS. Results also indicated the need for guidelines on patient management for pregnant women with MS.

## 1. Introduction

Management of multiple sclerosis (MS) and decision-making surrounding pregnancy are complex subjects [1] which includes the risk of relapse during pregnancy and post-partum, the use of disease modifying therapies (DMTs) before/during pregnancy and breastfeeding, and the impact of breastfeeding on the course of MS. For years, pregnancy was believed to have a negative effect on multiple sclerosis [1], and while this has not been supported by evidence, women with MS continue to report experiencing negative attitudes about pregnancy and MS from their healthcare providers, often reporting feeling pressured into making a decision regarding child-bearing prior to the age of 30 or before their MS has progressed [2].

There has been a well-documented decrease in MS relapses during pregnancy [1, 3–5]; however the risk of relapse post-partum increases significantly for women with MS [1, 5]. DMTs have become a critical strategy to control the course of MS. While there is emerging data on the use of DMTs during pregnancy for women with MS, this information remains limited in size and scope [6–10] and formal studies are missing, for obvious reasons. Analyses of data on whether or not breastfeeding reduces the risk of MS relapse provide mixed conclusions [3–5, 11–14] and there is little to no empirical data on the impact of the use of DMT during breastfeeding [15]. Extended periods off DMTs though are contrary to general recommendations of early treatment with DMT and may potentially have a negative impact on women's MS disease.

The purpose of this study was to examine what specific advice healthcare providers (HCPs) typically offer patients with MS who are planning on becoming pregnant, are currently pregnant, or have given birth. An anonymous survey was developed and administered to HCPs who work with individuals with MS in various clinical settings.

## 2. Materials and Methods

The survey was distributed to seventy-two HCPs in the United States who were attending the 6th Annual Regional Multiple Sclerosis Summit in Seattle, WA, in May 2011. Twenty-eight returned completed surveys, resulting in a response rate of 39%. The National Multiple Sclerosis Society sponsored this regional, one-day, continuing medical education conference on clinical MS management. Attendees were invited to participate in an anonymous survey on clinical practices regarding their experience with and management of pregnancy in MS. The 66-question survey was developed by the first and third authors, one an MS-fellowship trained neurologist and the other a health outcomes researcher with training in measurement. The questions were formulated using the current literature on pregnancy and breastfeeding in MS including a prior related study on this topic [16] as well as professional experience. The survey was revised based on feedback by several physicians experienced in treating MS patients. Participating HCPs were asked about their recommendations regarding pregnancy, breastfeeding, and post-partum periods for women with MS, focusing on how these topics relate to DMTs. The Institutional Review Board of the University of Washington approved all procedures. To analyze the data from this survey, summary statistics were calculated and are presented in the following section.

## 3. Results and Discussion

Twenty-six of the 28 healthcare providers surveyed for this study provided demographic information. Over half of the sample was female, and a majority (*n* = 18, 64%) were between the ages of 41 and 60. The majority of respondents were medical doctors (*n* = 20, 72%), three-fourths of whom were neurologists (*n* = 15). About one-third of each had been in practice for less than 10 years (*n* = 10, 36%) or 11 to 20 years (*n* = 8, 29%), and their most common practice setting was in private practice (*n* = 12, 43%). Participants reported a considerable MS patient load, with 58% (*n* = 16) seeing more than 5 MS patients per week and with one-fourth (*n* = 7, 25%) seeing between one and five MS patients per week, one participant (4%) seeing one MS patient per week and four (14%) not reporting.

In response to questions about their approach to family planning when working with women with MS, less than half of the healthcare providers (*n* = 13, 46%) encouraged pregnancy if a patient desired it, but almost all (*n* = 27, 97%) reported that they would not discourage pregnancy because of a patient's MS diagnosis. Less than one-fifth (*n* = 5, 18%) of the healthcare providers reported that their recommendations regarding pregnancy might depend on their patient's clinical course of MS.

When asked about their recommendations regarding DMT, over half of the participants (*n* = 16, 57%) reported that they always and 29% (*n* = 8) that they sometimes initiate a discussion about family planning when women of childbearing age begin taking DMTs. Nearly two-thirds (*n* = 17, 61%) always recommend that patients stop DMT prior to pregnancy, that is, discourage use of DMTs for patients desiring to conceive. About one-third (*n* = 10, 36%) reported recommending that patients stop taking DMT as soon as they find out that they are pregnant. For some (*n* = 5, 18%), these recommendations depended on which DMT the patient was taking. Five HCPs (18%) reported that the patient's clinical course dictated whether or not they would recommend ceasing DMTs.

Less than half of the HCPs surveyed (*n* = 12, 43%) recommended that patients not spend long periods of time without taking DMTs while trying to become pregnant. Finally, 10 of the HCPs surveyed (36%) reported that they had at least one patient with MS become pregnant while on a DMT they considered potentially harmful to the fetus. See Table 1.

Thus, while previous reports indicated that women with MS report receiving negative messages about pregnancy and MS [2], HCPs in this study overwhelmingly reported that they are supportive or even encouraging of women with MS wanting to have children. It is possible that the lack of active encouragement by the HCP might be perceived by women with MS as lack of support of their desire to become pregnant.

Most HCPs recommended that women stop using DMT prior to becoming pregnant, or at least once they have conceived, which is consistent with previous research [16]. For many of these HCPs, recommendations depended both on the clinical course of MS as well as which DMT the patient was taking. Still, these results suggest that the HCPs recommendations vary considerably. These inconsistent recommendations probably reflect the limited evidence related to the effects of DMT in regards to pregnancy. Most data is available for glatiramer acetate (pregnancy category B) and interferon beta 1a/1b (pregnancy category C) [7, 8, 10]. Concerns of the use of interferon beta 1a/1b leading to an increased risk of spontaneous abortion are not supported by data from several registries [7, 8] and it has been found to be safe in follow-up studies when used for up to 4 weeks after conception [9]. Limited human pregnancy outcome data is available for natalizumab (pregnancy category C) [6] and even less for the newer, oral agents which raise concern regarding their potential for greater placental transmission and because of the existence of some negative animal data (fingolimod and dimethyl fumarate pregnancy category C and terflunomide pregnancy category X) [7].

Over half of the participants (*n* = 15, 54%) reported that they discuss breastfeeding risks and benefits with their patients, and nearly two-thirds (*n* = 17, 61%) reported that they inform patients about the increased risk of relapse during the post-partum period.

Three-fourths of the HCPs (*n* = 21, 75%) inform their patients that DMTs are not recommended during breastfeeding. No one reported that they would counsel against breastfeeding due to the risk of relapse during the post-partum period, yet two HCPs (7%) would recommend foregoing breastfeeding in order to start taking DMTs. Twenty-five percent (*n* = 7) advised that patients breastfeed, believing that benefits likely outweigh the risk of relapse. A majority (*n* = 19, 68%) of the HCPs did not support breastfeeding as long as possible though, likely to limit the women's time off DMTs. Questions specific to recommended duration of breastfeeding were not asked.

Over one-third of HCPs (*n* = 11, 39%) discussed with their patients that breastfeeding may decrease the risk of post-partum relapse. About a third of the sample (*n* = 9, 32%) reported that their recommendations regarding breastfeeding depended on the clinical course of the disease, that is, suggesting that patients limit breastfeeding if they have a more aggressive disease course. Eighteen percent of the HCPs (*n* = 5) may recommend starting DMT despite breastfeeding due to the increased risk of relapses, and 21% (*n* = 6) actually advise that their patients restart DMT because they believe that the risk of relapse outweighs the potential risks associated with breastfeeding while taking DMTs. The majority of HPCs (*n* = 21, 75%) typically recommended restarting DMT after breastfeeding while 21% (*n* = 6) recommended that DMTs be resumed immediately after pregnancy regardless of whether or not the patient was breastfeeding.

Thus, overall, the HCP response to breastfeeding was positive, with most recommending that women with MS breastfeed but perceptions of risk/benefit assessments varied. This may be due to the inconsistent data concerning the effect of breastfeeding on MS relapse and insufficient data regarding the risks of DMTs during lactation. Some studies found a reduced post-partum relapse rate with exclusive breastfeeding for 2 months, though other studies fail to support this correlation [3–5, 11–14]. Additionally, HCPs preferred to have the discussion about breastfeeding with their patients, as opposed to referring them to their OBGYN or primary care physician (*n* = 24, 86%). This could be related to concerns that those providers may not be prepared to discuss the benefits and risks of DMT use, breastfeeding, and MS disease activity in the post-partum setting [15].

Healthcare providers were asked about the importance of resources that can help with decision-making. Results are presented in Table 2. Most indicated that FDA product labeling and recommendations from other neurologists/colleagues were important sources of information for the management of MS during pregnancy and/or breastfeeding compared to animal data, case reports, and postmarketing pregnancy registries which are held by pharmaceutical companies. The personal experience of the HCP was found to be a “somewhat important” resource. Finally, most HCPs noted that there are no evidence-based guidelines for patient management of pregnancy and breastfeeding. Development and dissemination of these guidelines would be useful for clinical practice.

## 4. Conclusions

The results of this study document the need for evidence-based recommendations related to the effect of DMT on pregnancy and breastfeeding, as well as the need for better guidelines on MS management related to family planning, pregnancy, and breastfeeding. While a randomized study of pregnant women taking DMTs is not to be expected in the future for ethical reasons [15], the independent and consistent use of a public registry of women with MS who become pregnant while taking DMTs or who utilized DMTs while breastfeeding would be beneficial as it could lead to more information about the effect of DMT use during pregnancy and breastfeeding. In the interim, providers may find a recently published review [7] on updated reproductive safety information from available preclinical and clinical data for DMTs helpful in guiding decision-making in this critical area of MS management.

Research on what advice HCPs give women with MS about pregnancy, breastfeeding, and DMT is limited. This study included a small sample, possibly nonrepresentative of the HCPs treating women with MS. As a result, the generalizability of the results is limited. Nevertheless, the study documents the need for more research and better evidence-based guidelines that HCPs need in order to provide the best care to their patients with MS who are pregnant or planning on conceiving.

## Figures and Tables

**Table 1:** DMT recommendations.

	Frequency	Percent
I initiate a discussion about family planning when I start women of child-bearing age on DMTs		
Always	16	57%
Sometimes	8	28%
Never	1	4%
Missing	3	11%
I always recommend that patients stop DMT prior to pregnancy		
No	10	36%
Yes	17	61%
Missing	1	3%
I always recommend that patients stop any DMT when conceived		
No	17	61%
Yes	10	36%
Missing	1	3%
I only recommend that patients stop DMT prior to pregnancy if on certain DMTs		
No	22	79%
Yes	5	18%
Missing	1	3%
My recommendations on when to stop DMT may depend on the patient's clinical course		
No	23	82%
Yes	5	18%
Missing	0	0%
I recommend that patients avoid long time periods without DMT while attempting pregnancy		
No	16	57%
Yes	12	43%
Missing	0	0%
I have had an MS patient who became pregnant on a DMT I consider potentially harmful to the fetus		
No	15	54%
Yes	10	36%
Missing	3	10%

**Table 2:** Sources of information for patient management during pregnancy.

	Frequency	Percent
I find FDA product labeling to be…in helping me with patient management during pregnancy and/or breastfeeding		
Not very important	2	7%
Somewhat important	5	18%
Very important	7	25%
Extremely important	11	39%
Missing	3	11%
I find animal data to be…in helping me with patient management during pregnancy and/or breastfeeding		
Not very important	1	4%
Somewhat important	10	36%
Very important	7	25%
Extremely important	6	21%
Missing	4	14%
I find case reports to be…in helping me with patient management during pregnancy and/or breastfeeding		
Not very important	2	7%
Somewhat important	7	25%
Very important	9	32%
Extremely important	5	18%
Missing	5	18%
I find postmarketing pregnancy registries to be…in helping me with patient management during pregnancy and/or breast feeding		
Not very important	1	4%
Somewhat important	8	29%
Very important	6	21%
Extremely important	10	36%
Missing	3	11%
I find personal experience to be…in helping me with patient management during pregnancy and/or breastfeeding		
Not very important	1	4%
Somewhat important	12	43%
Very important	6	21%
Extremely important	6	21%
Missing	3	11%
I find the recommendations of other neurologist/colleagues to be…in helping me with patient management during pregnancy and/or breastfeeding		
Not very important	1	4%
Somewhat important	5	18%
Very important	14	50%
Extremely important	5	18%
Missing	3	11%
I'm not aware of any evidence-based guidelines for patient management of pregnancy and breastfeeding		
No	20	71%
Yes	6	21%
Missing	2	7%
